# Prevalence and Genomic Insight of Extended-Spectrum β-Lactamase-Producing *Enterobacteriaceae* (ESBL-E) Isolated from Food Sources in Saudi Arabia

**DOI:** 10.3390/foods15152585

**Published:** 2026-07-23

**Authors:** Shahad Alsubaie, Amani T. Alsufyani, Norah Alotaibi, Ashwaq Alhamed, Shahad Alsalman, Manal Almusa, Ahmad Aljohani, Amal Sabour, Lenah Mukhtar

**Affiliations:** 1 College of Science, King Saud University, Riyadh 11451, Saudi Arabia; shahad.alsubaie1@hotmail.com; 2 Saudi Food and Drug Authority (SFDA), Riyadh 11671, Saudi Arabia; atsufyani@sfda.gov.sa (A.T.A.); nmaotaibi@sfda.gov.sa (N.A.); as.hamed@sfda.gov.sa (A.A.); shahad.alsalman@outlook.com (S.A.); mamusa@sfda.gov.sa (M.A.); ahmad.90.aljohani@gmail.com (A.A.)

**Keywords:** extended-spectrum β-lactamase (ESBL), *Enterobacteriaceae*, foodborne bacteria, antimicrobial resistance, *Salmonella* Infantis, whole-genome sequencing, food safety surveillance, Saudi Arabia

## Abstract

*Enterobacteriaceae* are a diverse family of Gram-negative, rod-shaped, facultatively anaerobic bacteria that serve as important indicators of food safety. Many species within this family, including common foodborne pathogens, are major contributors to both community- and hospital-acquired infections. The widespread use of antibiotics in agriculture has facilitated the emergence and dissemination of antimicrobial-resistant bacteria in food products, livestock, and the environment. This study investigated the prevalence and genomic characteristics of extended-spectrum β-lactamase (ESBL)-producing *Enterobacteriaceae* isolated from food sources in Saudi Arabia. A total of 160 isolates preserved in the Saudi Food and Drug Authority (SFDA) biobank were screened for antimicrobial resistance using indicator β-lactam antibiotics according to CLSI guidelines. Phenotypic screening indicated that *Escherichia coli* O145, *E. coli* O157, *Klebsiella pneumoniae*, and *Cronobacter sakazakii* isolates were not ESBL producers. In contrast, ESBL production was detected among *Salmonella* spp. isolates, and 13 confirmed ESBL-producing *Salmonella* isolates were subsequently subjected to whole-genome sequencing (WGS). Genomic analysis identified the ESBL gene *blaCTX-M-65*, along with multiple antimicrobial resistance determinants, virulence-associated genes, and plasmid replicons, predominantly belonging to the IncFIB and IncX families. These findings provide important insights into the occurrence of ESBL-producing *Enterobacteriaceae* in food sources and highlight the value of integrating phenotypic and genomic approaches for antimicrobial resistance surveillance within food safety systems.

## 1. Introduction

Members of the family *Enterobacteriaceae* are a diverse group of Gram-negative, rod-shaped, facultatively anaerobic bacteria that inhabit humans, animals, food, and environmental niches [1]. Many species are capable of fermenting carbohydrates into a variety of end products and are widely used as microbiological indicators for assessing food safety and hygiene practices [1]. However, some genera, such as *Escherichia*, *Klebsiella*, *Proteus*, and *Salmonella* spp., are recognized as important foodborne pathogens responsible for gastrointestinal infections, hospital outbreaks, and community-acquired disease [1]. The dual role of *Enterobacteriaceae* as both hygiene indicators and pathogenic organisms highlights their importance in food production systems and public health surveillance.

The rise in antimicrobial resistance (AMR) in *Enterobacteriaceae* has become a major global concern. The widespread use of antibiotics in human healthcare, veterinary medicine, and agricultural production has created strong selective pressure that promotes the emergence and spread of resistant bacteria [2]. In addition, antimicrobial residues present in livestock, aquaculture, crops, and water systems facilitate the persistence and dissemination of resistant strains across ecological boundaries [2]. This has serious implications for food safety, since resistant bacteria can enter the food chain, survive processing, and ultimately pose risks to humans. The World Health Organization (WHO) has recognized AMR as a significant threat to global health, food security, and sustainable development [3].

The production of extended-spectrum β-lactamases (ESBLs) is especially significant among the resistance mechanisms observed in *Enterobacteriaceae*. These enzymes confer resistance to penicillins, aztreonam, and the first-, second-, and third-generation cephalosporins, resulting in limited therapy alternatives [4]. Traditionally, carbapenems have been the preferred treatment for ESBL-producing *Enterobacteriaceae* (ESBL-E); however, the rising prevalence of carbapenemase-producing strains now threatens the effectiveness of this last-resort therapy [4]. Genes that encode extended-spectrum β-lactamases (ESBLs), particularly *blaCTX-M-65*, *blaSHV*, and *blaTEM*, have become globally widespread and are commonly detected in clinical isolates as well as in food, animals, and environmental reservoirs [5]. This widespread distribution highlights the food chain as an important pathway for the dissemination of ESBL-producing bacteria.

The presence of ESBL-producing *Enterobacteriaceae* in food sources is a significant global public health risk [4]. These bacteria can spread along the food chain, from agricultural settings, through food processing, and finally to consumers, where they can cause infections that are difficult to treat due to antimicrobial resistance [4]. ESBL enzymes render third-generation cephalosporins ineffective, which are critical for the treatment of invasive infections, thereby further limiting available therapeutic options [4].

The worldwide impact of ESBL-E has become increasingly important. It is anticipated that over one billion individuals may be infected globally, with the largest prevalence observed in Asia and the Middle East [6,7]. Notably, food products including chicken, beef, dairy, and fresh produce have consistently been identified as potential vehicles for the transmission of ESBL-E to humans [8].

This highlights the necessity for surveillance systems that incorporate the One Health approach, connecting human, animal, and environmental health to mitigate the spread of antimicrobial resistance via food systems [9].

Despite increasing global interest, data regarding the prevalence of ESBL-producing *Enterobacteriaceae* in food sources in Saudi Arabia remains limited. Most studies conducted in Saudi Arabia have focused on clinical isolates, whereas investigations involving food have generally been restricted to specific bacterial species, food commodities, or geographical locations [10,11,12,13,14,15]. Consequently, the occurrence and distribution of ESBL-producing *Enterobacteriaceae* across different food sources remains insufficiently characterized. Furthermore, limited information is available linking phenotypic antimicrobial resistance with ESBL genes, additional antimicrobial resistance determinants, sequence types, virulence-associated genes, and plasmid replicons among foodborne isolates. This lack of integrated epidemiological and genomic evidence limits the identification of important food reservoirs and hinder assessment of the potential dissemination of ESBL-producing bacteria and their associated resistance determinants through the Saudi food chain [16,17,18]. Given the Kingdom’s extensive food market, significant domestic production, and continued reliance on imported food products, addressing these knowledge gaps is essential to strengthen evidence-based risk assessment, antimicrobial resistance surveillance, and targeted food-safety interventions.

Conventional phenotypic techniques, such as disk diffusion and broth microdilution, are crucial for identifying resistance; nevertheless, they offer limited insight into the genetic background, diversity, and movement of resistance determinants [19]. Conversely, whole-genome sequencing (WGS) has become a fundamental tool in antimicrobial resistance (AMR) research [20]. WGS facilitates an exhaustive investigation by concurrently detecting resistance genes, virulence factors, plasmid replicons, and sequence types with exceptional precision [20]. In addition, WGS provides strain-level resolution, enabling the distinction of clonal lineages and the monitoring of epidemic clones, such as *Salmonella* Infantis ST32 [21]. When complemented by dedicated analyses of chromosomes, plasmids, and other mobile genetic elements, WGS can further elucidate the genomic context of resistance determinants and improve understanding of their potential dissemination across the food–animal–human interface [22]. In the present study, WGS was used to detect antimicrobial resistance genes, virulence-associated genes, plasmid replicons, and sequence types. However, the chromosomal or plasmid location of these genes and their transferability were not investigated.

Therefore, this study aimed to investigate the occurrence, resistance profiles, and genetic determinants of ESBL-producing *Enterobacteriaceae* isolated from food products sampled in Saudi Arabia. Phenotypic antimicrobial susceptibility testing was integrated with whole-genome sequencing to characterize ESBL genes, associated antimicrobial resistance determinants, virulence-associated genes, plasmid replicons, and bacterial sequence types.

The contribution of this study does not lie in identifying a previously undescribed genotype or transmission pathway. Rather, it lies in integrating phenotypic ESBL screening of a multi-species, multi-food-source surveillance collection with genomic characterization of the confirmed ESBL-producing isolates. This approach enabled assessment of whether clinically important resistance was broadly distributed across the examined food isolates or concentrated within particular bacterial lineages and food sources. The findings provide baseline evidence to support risk-based food-chain antimicrobial resistance surveillance and the prioritization of resistant lineages for further genomic and epidemiological investigation within a One Health framework.

## 2. Materials and Methods

### 2.1. Study Design, Bacterial Isolates, and Source

This retrospective, laboratory-based study included (160 non-duplicate *Enterobacteriaceae* isolates preserved in the Saudi Food and Drug Authority (SFDA) Reference Laboratory for Microbiology Biobank. The isolates were previously recovered and identified from food samples through routine national food safety surveillance programs conducted by the SFDA, following standardized ISO microbiological methods.

The isolates were recovered between 2020 and 2023 from food samples collected from multiple cities in Saudi Arabia. To maintain the confidentiality of routine surveillance data, geographic information is presented in aggregate to rather than at the individual sampling-site level. The isolates consisted of six groups: *Escherichia coli O157* (*n* = 68), *Escherichia coli* O145 (*n* = 5), *Salmonella* spp. (*n* = 56), *Klebsiella pneumoniae* (*n* = 15), *Proteus mirabilis* (*n* = 13), and *Cronobacter sakazakii* (*n* = 3). All isolates were recovered from chicken meat, beef meat, leafy vegetables, and powdered milk, as summarized in Table 1. Before antimicrobial susceptibility testing and genomic DNA extraction, each isolate was revived on nutrient agar (NA; Oxoid, Basingstoke, UK) and incubated aerobically at 37 °C for 24 h. Culture purity was assessed based on the presence of uniform, well-isolated colonies. A single colony was then selected for bacterial identification using matrix-assisted laser desorption/ionization time-of-flight mass spectrometry (MALDI-TOF MS; Bruker Daltonics GmbH & Co. KG, Bremen, Germany). All eligible isolates available in the SFDA Biobank during the study period were included; therefore, the number of isolates varied among bacterial species according to their recovery through routine food-safety surveillance. Each isolate originated from a separate food sample and was analysed independently for antimicrobial susceptibility and genomic characteristics.

### 2.2. Antimicrobial Susceptibility Testing (AST) and ESBL Confirmation

Initial screening for possible ESBL production was performed by using the disk diffusion method according to the Kirby–Bauer test protocol in accordance with Clinical and Laboratory Standards Institute (CLSI) (M100-34th Edition) guidelines [23,24]. Briefly, bacterial suspensions were adjusted to a 0.5 McFarland standard and inoculated onto Mueller–Hinton agar (Condalab, Madrid, Spain) plates. Antibiotic disks (Oxoid, Basingstoke, UK) including ceftazidime (CAZ, 30 μg), cefotaxime (CTX, 30 μg), ceftriaxone (CRO, 30 μg), aztreonam (ATM, 30 μg), and cefpodoxime (CPD, 10 μg) were applied for all strains except for *Proteus* isolates as the azterionam was not tested. Then the plates were incubated at 37 °C for 18–24 h. The diameters of inhibition zones were measured and interpreted according to CLSI breakpoints (M100-34th Edition) [24]. *Escherichia coli* ATCC^®^ 25922 was used as a quality control strain.

Confirmation of possible ESBL was conducted using the Sensititre™ system (Thermo Fisher Scientific, Waltham, MA, USA), which applies the Broth Microdilution (BMD) method in accordance with CLSI guidelines (M100). The EUVSEC2 AST panel was used, which is specifically designed for ESBL detection in Gram-negative bacteria following the manufacturer’s instructions. Briefly, a single colony was suspended in 5 mL sterile demineralized water (Cat. No. T3339, Thermo Fisher Scientific, Waltham, MA, USA) to reach a 0.5 McFarland standard (10^7^–10^8^ CFU/mL) using a Sensititre™ Nephelometer. A 10 µL aliquot was inoculated into 11 mL Mueller–Hinton broth (MHB; Cat. No. T3462, Thermo Fisher Scientific, Waltham, MA, USA), mixed, and dispensed (50 µL per well) into Sensititre plates using the AIM™ Automated Inoculation Delivery System (Thermo Fisher Scientific). Plates were sealed, incubated at 37 °C for 18–24 h, and read with a Sensititre™ OptiRead™ (Thermo Fisher Scientific). After reading, the minimum inhibitory concentrations (MICs) were interpreted using Sensititre SWIN™ software according to CLSI breakpoints (M100-34th Edition) [24]. ESBL production was confirmed when the MIC of ceftazidime or cefotaxime decreased by ≥3 two-fold dilutions in the presence of clavulanic acid compared to the antibiotic alone [24].

The EUVSEC2 panel also included cefepime, cefoxitin, and temocillin, enabling detection of resistance beyond typical ESBL activity (e.g., AmpC and OXA-48-like enzymes). Carbapenems (ertapenem, imipenem, meropenem) were also tested to assess the co-existing of carbapenemase activity. The quality control strains were *Klebsiella pneumoniae* ATCC^®^ 700603 (ESBL positive), *Escherichia coli* ATCC^®^ 25922 (ESBL negative), and *Pseudomonas aeruginosa* ATCC^®^ 27853, and they were included in every test run.

### 2.3. Genomic DNA Extraction

Genomic DNA was extracted using the DNeasy Blood & Tissue Kit (Qiagen, Hilden, Germany) according to the manufacturer’s instructions. Briefly, single colonies grown overnight at 37 °C were suspended in 1 mL sterile distilled water, centrifuged, and lysed with ATL buffer and proteinase K, followed by AL buffer and ethanol precipitation. DNA was bound to a spin column, washed with AW1 and AW2 buffers, and eluted in 50 µL AE buffer. DNA was stored at −20 °C until further use.

### 2.4. Whole-Genome Sequencing (WGS)

DNA purity was verified by A260/A280 ratio (≥1.8) using a QIAxpert spectrophotometer (Qiagen, Hilden, Germany) and quantified with a QFX Fluorometer (Denovix, Wilmington, DE, USA). Libraries were prepared with the Nextera XT kit (Illumina, San Diego, CA, USA), purified with AMPure XP magnetic beads (Beckman Coulter, Brea, CA, USA), normalized, and pooled. Sequencing was performed on an Illumina MiSeq platform using 600-cycle reagent kits (v3) with 5% PhiX spike-in. Adapter trimming and demultiplexing were per-formed using bcl2fastq (v2.20). Data quality was assessed with Quast (v4.6.3).

### 2.5. Bioinformatics Analyses

Initial quality control of raw sequencing reads was performed using FastQC (v0.12.1) and summarized using MultiQC (v1.31). Read trimming and quality filtering were performed using Trimmomatic (v0.39). De novo genome assembly was carried out using SPAdes (v3.15.2), followed by assembly quality assessment using QUAST (v5.0.2). The resulting draft assemblies were used for downstream analyses, including species identification, multilocus sequence typing, serotype prediction, antimicrobial resistance gene detection, virulence-associated gene identification, and plasmid replicon detection. All analyses were performed within the TORMES pipeline framework (v1.3.0). Isolates were taxonomically classified using the RDP Classifier (v2.0.3). Multilocus sequence typing was conducted using MLST (v2.19.0) to determine sequence types (STs) and identify the most common strain lineages and serotypes. Antimicrobial resistance genes (ARGs) were identified using CARD (v4.0.0) with a nucleotide identity threshold of ≥90% and a minimum coverage of ≥60%. Plasmid replicon types were detected using PlasmidFinder (v2.1) with a minimum identity threshold of 95% and coverage of 60%. Virulence factors were identified using the Virulence Factor Database (VFDB) applying cutoff values of ≥90% identity and ≥50% coverage. Default parameters were used for all bioinformatics software tools unless otherwise stated.

### 2.6. Statistics and Data Visualization

This study was descriptive in nature. Categorical variables were summarized as frequencies and percentages. No inferential statistical analyses or hypothesis-testing procedures were performed because the study was designed to characterize the prevalence and genomic features of archived bacterial isolates rather than compare experimental groups.

All figures and data visualizations were generated using R (R Core Team, 2022) from CSV files prepared after bioinformatics processing. Heatmaps, including AMR profiles, antimicrobial resistance gene (ARG) distributions, plasmid replicon types, and virulence gene category profiles, were produced using custom R scripts. Prevalence of antimicrobial resistance phenotypes, ARGs, plasmids, and virulence genes was calculated as percentages based on the total number of isolates.

## 3. Results

### 3.1. Sample Sources and Isolate Distribution

A total of 160 non-duplicate *Enterobacteriaceae* isolates from food sources were included in the study. The distribution of the isolates according to bacterial group and food source is summarized in Table 1.

### 3.2. Phenotypic Screening of ESBL Production

Initial screening for extended-spectrum β-lactamase (ESBL) production was performed using the disk diffusion method with indicator β-lactam antibiotics, including aztreonam, cefotaxime, cefpodoxime, ceftriaxone, and ceftazidime. All *Escherichia coli* O157 (*n* = 68), *E. coli* O145 (*n* = 5), *Cronobacter sakazakii* (*n* = 3), and *Klebsiella pneumoniae* (*n* = 15) isolates were susceptible to all tested antibiotics, and no resistance was detected during the initial ESBL screening.

In contrast, resistance to at least one of the indicator antibiotics was detected in all *Proteus mirabilis* isolates (13/13, 100%) and in the majority of *Salmonella* spp. isolates (53/56, 94.6%) during the initial ESBL screening (Table 2).

### 3.3. Phenotypic Confirmation of ESBL Production

**Figure 1:**
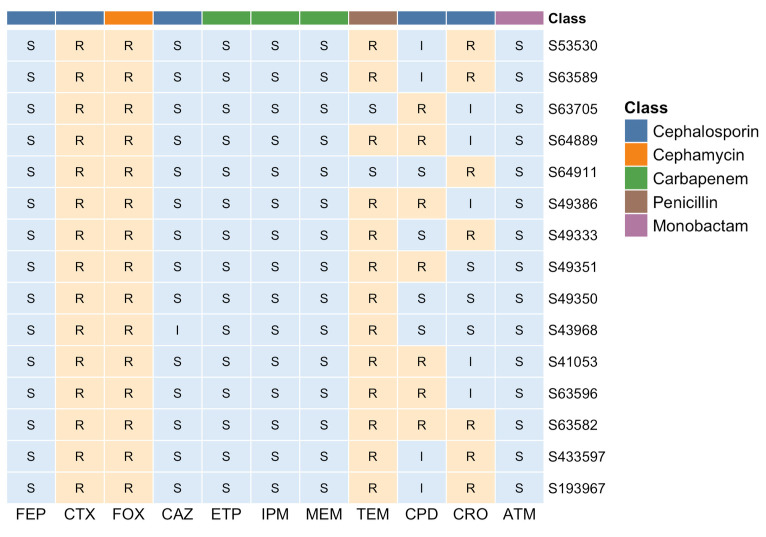
Antimicrobial susceptibility profiles of phenotypically confirmed ESBL-producing Salmonella isolates. The heatmap showing antimicrobial susceptibility testing (AST) results for the 15 Salmonella isolates confirmed as ESBL producers. Susceptibility interpretations are indicated as susceptible (S), intermediate (I), or resistant (R) according to CLSI breakpoints. Antibiotics are cephalosporins cefepime (FEP), cefotaxime (CTX), cefoxitin (FOX), ceftazidime (CAZ), ertapenem (ETP), imipenem (IPM), meropenem (MEM), temocillin (TEM), cefpodoxime (CPD), ceftriaxone (CRO), and aztreonam (ATM). These antibiotics belong to the following antimicrobial classes: cephalosporins (FEP, CTX, CAZ, CPD, CRO), cephamycin (FOX), carbapenems (ETP, IPM, MEM), penicillin (TEM), and monobactam (ATM).

Antimicrobial susceptibility testing (AST) was performed to the ESBL-positively screened isolates using the broth microdilution method according to CLSI guidelines. These isolates demonstrated resistance to several β-lactam antibiotics, particularly third generation cephalosporins, including cefotaxime and ceftriaxone. Resistance to cefoxitin was also observed among several isolates. In contrast, all isolates remained susceptible to carbapenems, including ertapenem, imipenem, and meropenem. Most isolates also retained susceptibility to cefepime and aztreonam, although variable responses were observed for cefpodoxime. The AST profiles of the isolates that were phenotypically confirmed as ESBL producers are summarized in Figure 1.

Phenotypic confirmation of ESBL production was performed according to CLSI confirmatory testing criteria using cefotaxime and ceftazidime tested alone and in combination with clavulanic acid. ESBL production was confirmed when a ≥3 two-fold reduction in MIC was observed in the presence of clavulanic acid compared with the antibiotic alone. Using these criteria, ESBL production was confirmed in 15 *Salmonella* isolates, while no ESBL production was detected among the other screened isolates. All confirmed isolates showed a ≥3 two-fold reduction in cefotaxime MIC in the presence of clavulanic acid, consistent with the ESBL phenotype. Ceftazidime MIC reductions were also observed and ranged from 4- to 16-fold. Detailed MIC values and fold-reduction calculations are provided in Supplementary Table S1. Overall, 94 of the 160 isolates (58.8%) did not exhibit phenotypic resistance to the antimicrobial agents tested. Antimicrobial resistance, including the ESBL phenotype, was concentrated in a limited number of isolates, particularly poultry-associated *Salmonella* Infantis.

### 3.4. Serotyping and MLST of Salmonella Isolates

Whole genome sequencing (WGS) was performed for all phenotypically confirmed ESBL-producing *Salmonella* isolates (*n* = 15). However, after quality control and genome assembly assessment, 13 genomes passed quality filtering and were retained for downstream genomic analyses. The correspondence between the laboratory isolate identifiers and the deposited sequencing records is provided in Supplementary Table S2. In silico serotyping identified all sequenced isolates as *Salmonella enterica* serovar Infantis. The 13 sequenced *Salmonella* Infantis isolates originated from food samples collected from multiple cities and during different years between 2020 and 2023, indicating that they were not restricted to a single geographic location or sampling year. MLST was performed using the *Salmonella enterica* MLST database and revealed that all 13 genomes belonged to sequence type ST32, which is consistent with the *Salmonella* Infantis serotype.

### 3.5. Detection of Antimicrobial Resistance Genes

**Figure 2:**
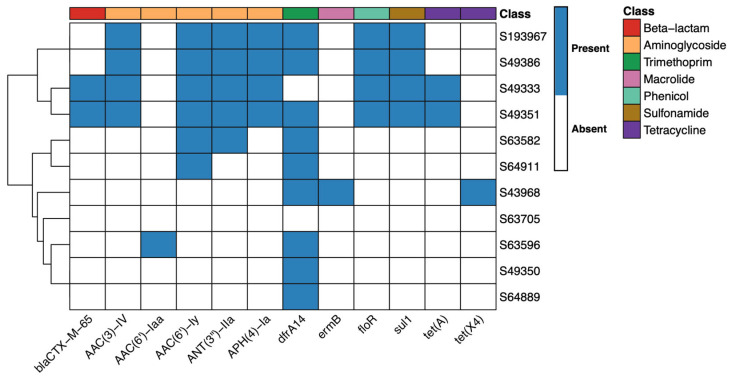
Distribution of acquired antimicrobial resistance genes among *Salmonella enterica* serovar Infantis isolates. The heatmap showing the presence and absence of acquired antimicrobial resistance genes identified using the Comprehensive Antibiotic Resistance Database (CARD) among the 11 isolates carrying at least one acquired ARG. Blue cells indicate the presence of a gene, while white cells indicate its absence. Genes are grouped according to antimicrobial class, including β-lactams, aminoglycosides, trimethoprim, macrolides, phenicols, sulfonamides, and tetracyclines.

WGS data analysis of the 13 *Salmonella* Infantis genomes revealed that 11 isolates carried one or more acquired antimicrobial resistance genes (ARGs), while two isolates did not carry acquired ARGs under the applied identity and coverage thresholds (Figure 2).

The extended-spectrum β-lactamase gene *blaCTX-M-65* was detected in two isolates. The most frequently detected resistance determinant was the trimethoprim resistance gene *dfrA14*, identified in nine isolates.

Several aminoglycoside resistance genes were also detected, including *aac(3)-IV* (4/13), *aac(6′)-Iy* (6/13), *aph(4)-Ia* (4/13), and *ant(3″)-IIa* (5/13). Additional resistance determinants included the sulfonamide gene *sul1* (4/13), the phenicol resistance gene *floR* (4/13), and tetracycline resistance genes *tet*(*A*) (2/13) and *tet*(*X4*) (1/13).

The macrolide resistance gene *erm(B)* was detected in one isolate. The distribution of these acquired ARGs across isolates is illustrated in Figure 2, highlighting variability in acquired resistance-gene profiles among the sequenced isolates. Moreover, a broader CARD-based analysis of resistance-associated genes, including multidrug efflux pumps and regulatory elements, revealed the presence of several genes across the isolates, including *acrB*, *acrD*, *emrA*, *emrB*, *emrR*, *mdtB*, *mdtC*, *mdtK*, *msbA*, and *mdsA*. The complete CARD-derived resistance-associated gene profile is presented in Supplementary Figure S1.

### 3.6. Virulence Gene Profiles

**Figure 3:**
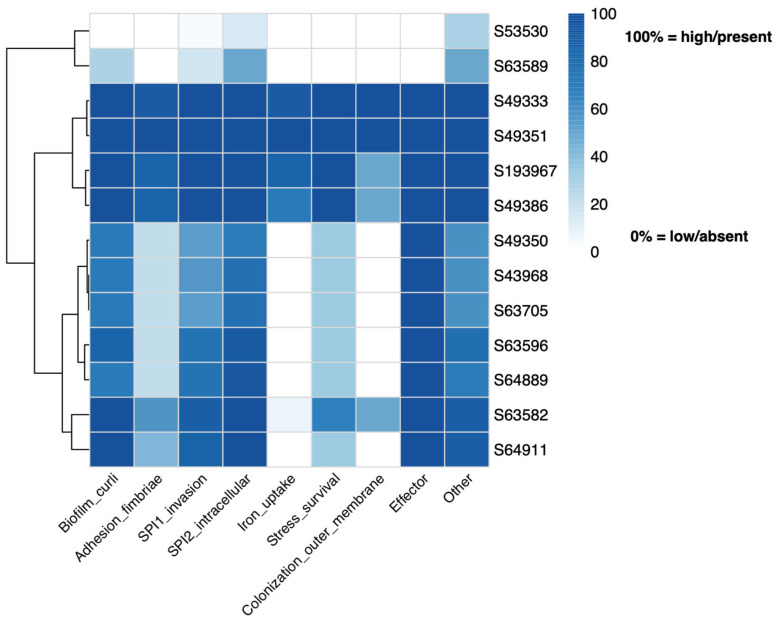
Virulence factor category profiles of *Salmonella enterica* serovar Infantis isolates. The heatmap showing the proportion of virulence genes detected in each functional category across the sequenced isolates based on whole genome sequencing analysis. Categories include biofilm formation, adhesion/fimbriae, invasion (SPI-1), intracellular survival (SPI-2), iron acquisition, stress survival, outer membrane colonization, effector proteins, and other virulence-associated determinants. Color intensity represents the percentage of virulence genes detected within each category for each isolate.

Whole genome analysis revealed the presence of multiple virulence-associated genes across the sequenced *Salmonella* Infantis isolates (Figure 3). These genes were associated with several functional categories involved in bacterial pathogenicity, including biofilm formation, adhesion and fimbriae, invasion, intracellular survival, iron acquisition, stress adaptation, outer membrane colonization, and effector proteins. Virulence determinants related to adhesion and fimbrial structures included genes involved in curli fimbriae, type I fimbriae, and long polar fimbriae, which contribute to bacterial attachment and colonization of host tissues. Genes associated with the *Salmonella* pathogenicity islands (SPI-1 and SPI-2) were also detected, including components of the type III secretion systems responsible for host cell invasion and intracellular survival. Additional virulence determinants included genes involved in iron acquisition systems, stress response mechanisms, and effector proteins that modulate host cell processes.

### 3.7. Plasmid Replicon Detection

**Figure 4:**
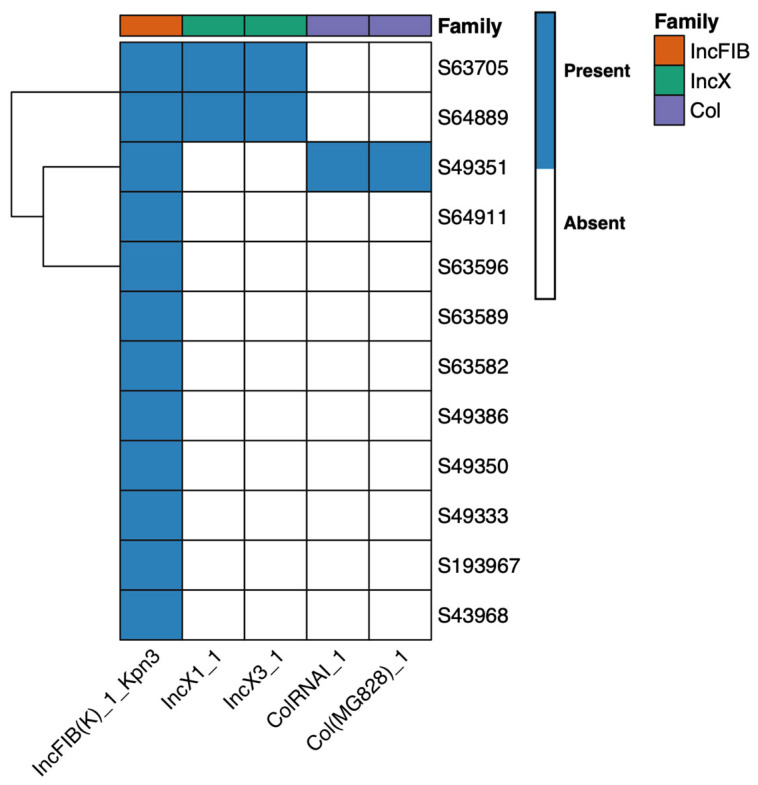
Plasmid replicon profiles of *Salmonella* Infantis isolates. The heatmap showing the presence and absence of plasmid replicon types identified from whole genome sequencing using in silico plasmid replicon detection. Blue cells indicate the presence of a replicon, while white cells indicate its absence. Replicons are grouped according to plasmid family, including IncFIB, IncX, and Col plasmids.

Plasmid replicon analysis was performed for the 13 *Salmonella* Infantis genomes that passed quality control and were retained for downstream genomic analysis. Plasmid replicons were detected in 12 out of 13 isolates. Five plasmid replicons belonging to three families were identified: IncFIB(K)_1_Kpn3, IncX1, IncX3, ColRNAI, and Col(MG828)_1. The IncFIB(K)_1_Kpn3 replicon was the most frequently detected and was present in the majority of isolates carrying plasmids (Figure 4). IncX-type plasmids, including IncX1 and IncX3, were identified in a subset of isolates. Col-type plasmids, including ColRNAI and Col(MG828)_1, were detected in a limited number of isolates. One isolate did not contain any detectable plasmid replicons.

## 4. Discussion

This study extends the available evidence on ESBL-producing *Enterobacteriaceae* in food sources in Saudi Arabia by integrating phenotypic ESBL confirmation with whole-genome sequencing. Among the 160 *Enterobacteriaceae* isolates representing multiple bacterial species and food categories, confirmed ESBL production was restricted to two poultry-associated *Salmonella enterica* serovar Infantis isolates. Genomic analysis of the draft genome assemblies identified these isolates as ST32 and demonstrated the co-occurrence of *blaCTX-M-65* with multiple antimicrobial resistance determinants, virulence-associated genes, and plasmid replicons appropriate for short-read genome assemblies.

The detected genotype and resistance determinants have been reported previously in poultry-associated *Salmonella* Infantis, including in Saudi Arabia [25,26]. Therefore, the present findings should be regarded as confirmation and extension of existing evidence rather than identification of a novel genotype or transmission pattern. The scientific contribution of the study lies in showing, within a broader multi-species food-surveillance collection, that clinically important ESBL production and multidrug resistance were not widely distributed among the examined isolates but were concentrated in a poultry-associated *Salmonella* Infantis ST32 lineage. This finding supports the prioritization of poultry-associated *Salmonella* Infantis for targeted surveillance and further high-resolution genomic investigation.

An epidemiologically important finding was that the majority of the examined *Enterobacteriaceae* isolates did not exhibit phenotypic antimicrobial resistance. This indicates that resistance was not broadly distributed across the bacterial species and food categories represented in the study collection. Instead, clinically important resistance, including the ESBL phenotype and multidrug resistance, was concentrated in a limited number of poultry-associated *Salmonella* Infantis isolates. This pattern suggests that resistance within the examined food isolates may be associated with particular bacterial lineages and food-production reservoirs rather than being uniformly disseminated throughout the collection. Accordingly, the subsequent genomic analysis focused on the ESBL-producing *Salmonella* Infantis isolates.

The detection of ESBL-producing *Salmonella* Infantis ST32 in the present study is consistent with reports describing the association of this lineage with poultry production, multidrug resistance, and extended-spectrum cephalosporin resistance [24,25,26,27,28,29]. In Saudi Arabia, previous studies have also confirmed the occurrence of *Salmonella* Infantis in poultry-production environments and retail chicken meat [30,31]. The agreement between these findings supports the importance of poultry as a potential reservoir of resistant *Salmonella* Infantis.

Nevertheless, differences in the reported frequency of ESBL-producing *Salmonella* Infantis among studies may reflect variation in sampling period, geographical coverage, food-source composition, sample size, bacterial isolation methods, and phenotypic confirmation criteria. Direct comparison should therefore be made cautiously.

The recovery of *Salmonella* Infantis isolates from multiple cities and different sampling years between 2020 and 2023 indicates that their occurrence within the surveillance collection was not restricted to a single location or time point. However, the available geographic and temporal metadata were insufficient to establish whether the isolates were epidemiologically linked or represented independent introductions. Although all sequenced isolates belonged to ST32, a shared sequence type alone does not confirm recent clonal transmission. Higher-resolution genomic analyses, such as core-genome multilocus sequence typing or single-nucleotide polymorphism analysis, together with supplier, establishment, production-lot, and detailed sampling metadata, would be required to investigate their genetic relatedness and possible epidemiological relationships [27,28,29].

Despite the presence of just two isolates harbouring the *blaCTX-M-65* gene, its identification is of considerable significance. The *blaCTX-M-65* gene has become increasingly linked to epidemic *Salmonella* Infantis strains and poses a significant public health threat due to its capacity to confer resistance to third-generation cephalosporins, which are among the primary antibiotics used for treating salmonellosis [4,5]. The detection of this gene was consistent with the phenotypic resistance observed in the corresponding isolates, supporting agreement between the genomic and antimicrobial susceptibility findings. The detection of this gene in *Salmonella* Infantis is consistent with findings from a previous study in Saudi Arabia, where *Salmonella* Infantis isolates from poultry carried *blaCTX-M-65* in 12 of 39 isolates [26]. This agreement suggests the continued occurrence of this resistance determinant among poultry-associated *Salmonella* Infantis in Saudi Arabia. However, the available data are insufficient to determine whether the isolates originated from a persistent local lineage or from repeated introductions through the poultry-production chain.

The presence of ESBL determinants alongside various acquired resistance genes in *Salmonella* Infantis from food sources may reflect the combined effects of antimicrobial selective pressure, persistence of successful resistant lineages, and dissemination of mobile genetic elements within poultry-production environments [30,32]. Evidence suggests extensive antibiotic use in poultry production, the significant prevalence of ESBL-producing *Enterobacteriaceae* in poultry farms in areas such as Makkah, and the identification of ESBL genes in *E. coli* from poultry meat in Saudi Arabia [15,31]. However, antimicrobial-use data were not collected in the present study; therefore, a direct relationship between antimicrobial exposure and the detected resistance profiles cannot be established.

All sequenced ESBL-producing *Salmonella* Infantis isolates carried multiple resistance genes, including those conferring resistance to aminoglycosides, tetracyclines, sulfonamides, trimethoprim, macrolides, phenicols, and peptide-associated resistance genes, as well as genes encoding efflux pumps. The multidrug resistance (MDR) pattern observed in our isolates is similar to findings reported in chicken production systems in Europe and South Korea, where *Salmonella* Infantis ST32 frequently harbors large plasmids containing multiple resistance genes [28]. The detection of genes such as *tet(A)*, *sul1*, and *dfrA14*, which are frequently linked to integrons, underscores the significance of mobile genetic elements in the dissemination of antimicrobial resistance [33]. The presence of resistance determinants affecting several antimicrobial classes within the same isolates may support co-selection of multidrug-resistant bacterial populations. However, the present analysis did not establish whether these genes were physically co-located on the same plasmid, integron, or other mobile genetic element. Nevertheless, confirmation that these genes are physically co-located on the same plasmid or other mobile genetic element would require complete plasmid reconstruction or long-read sequencing.

Although mobile genetic elements are recognized mechanisms for the horizontal dissemination of antimicrobial resistance, the present study did not perform conjugation experiments or other functional transfer assays. Therefore, no conclusion can be drawn from the current data regarding the horizontal transfer or conjugative transferability of the detected resistance determinants. This MDR profile is not only consistent with reports from Europe and South Korea, but also aligns with recent findings in Saudi Arabia, where *Salmonella* Infantis ST32 isolates from poultry carried *blaCTX-M-65* on IncFIB plasmids alongside additional genes such as *tet(A)*, *sul1*, and *dfrA14* [26].

Besides resistance, all isolates possessed a variety of virulence genes, including fimbrial adhesins (*csg*, *fim*, *lpf*), Type III secretion systems (SPI-1 and SPI-2 clusters), and iron acquisition systems (*ent*, *fep*, *ybt*). The presence of these genes indicates genomic potential for host adhesion, epithelial-cell invasion, intracellular survival, iron acquisition, and adaptation to stressful conditions. However, genomic detection confirms the presence of the genes but does not demonstrate their expression or biological activity under food-production or host-associated conditions.

The identification of *avrA*, which modulates host apoptosis, and *mig-14*, which provides resistance to antimicrobial peptides, further supports the hypothesis that these strains are particularly adapted for persistence in both the food production environment and the human host [34,35]. The detected virulence-gene profiles were broadly consistent with those reported in other poultry-associated and clinically relevant S. Infantis lineages [34,35].

Twelve isolates carried the IncFIB(K)_1_Kpn3 plasmid replicon, while additional replicons, including IncX1_1 and IncX3_1, were also identified. IncFIB-type plasmids have been widely reported as carriers of antimicrobial resistance and virulence-associated genes in *Enterobacteriaceae* [36,37]. In the present study, plasmid replicons and multiple antimicrobial resistance determinants were detected within the same isolates, However, this finding represents co-occurrence at the isolate level only. However, this finding represents co-occurrence at the isolate level only. Because contig-level gene localization and complete plasmid reconstruction were not performed, the physical location of individual resistance or virulence-associated genes on the detected plasmids could not be established. Therefore, their simultaneous detection should not be interpreted as evidence of direct genetic linkage, horizontal gene transfer, or conjugative transferability.

No carbapenemase genes were detected in the sequenced isolates. Accordingly, the presence of IncX replicons alone should not be interpreted as evidence of carbapenemase carriage. Dedicated genomic-context analysis, complete plasmid reconstruction, long-read or hybrid sequencing, and functional transfer experiments would be required to determine the genetic structure, resistance-gene content, and potential transferability of the detected plasmids.

In conclusion, the co-occurrence of multidrug resistance, ESBL production, and virulence factors in foodborne *Salmonella* Infantis isolates poses a significant threat to public health and food safety in Saudi Arabia. From a One Health perspective, these strains may circulate between food, animals, and humans, underscoring the necessity for comprehensive antimicrobial resistance surveillance.

From a food-industry perspective, the concentration of ESBL production within poultry-associated *Salmonella* Infantis supports risk-based surveillance across the poultry-production chain, including farms, hatcheries, slaughterhouses, processing facilities, and retail markets. Practical control measures should include strengthened sanitation verification, environmental monitoring, prevention of carcass and equipment cross-contamination, and maintenance of supplier and production-lot traceability.

The integration of antimicrobial susceptibility testing with whole-genome sequencing could support the early detection of *blaCTX*-M-65-positive ST32 lineages, improve source tracing during contamination events, and help distinguish persistent production-associated strains from sporadic introductions. The findings also reinforce the importance of prudent antimicrobial use in poultry production to reduce selective pressure favouring multidrug-resistant bacterial populations.

A limitation of this study is the relatively small number of analyzed isolates, including *Salmonella* Infantis, from which ESBL production was detected and sequenced. Although the results provide important preliminary evidence, a larger number of isolates would be required to support the conclusions and determine whether ST32 is the predominant ESBL clonal lineage in food in Saudi Arabia.

Another limitation relates to the draft nature of the genome assemblies. Assembly quality was evaluated using standard assembly statistics generated by the TORMES pipeline (Supplementary Report); however, dedicated genome-quality metrics such as completeness and contamination were not assessed using specialized tools (e.g., CheckM). Consequently, the assembled genomes should be regarded as draft assemblies rather than complete genomes. The genomic analyses presented in this study were therefore limited to applications appropriate for short-read draft assemblies, including species identification, multilocus sequence typing, serotype prediction, antimicrobial resistance gene detection, virulence-associated gene identification, and plasmid replicon detection. Analyses requiring complete genome reconstruction, including definitive gene absence, chromosomal versus plasmid localization, structural variation, or comprehensive comparative genomics, were beyond the scope of the present study and should be interpreted with caution. Future studies incorporating long-read or hybrid genome sequencing together with comprehensive genome-quality assessment will enable more complete genomic characterization.

The study was also based on an archived, non-random collection of isolates obtained through routine food-safety surveillance, with unequal representation of bacterial species and food categories. Therefore, the findings describe the isolates available in the SFDA Biobank, and neither the low frequency of ESBL production nor the high proportion of isolates without detected antimicrobial resistance should be interpreted as nationally representative estimates for foodborne *Enterobacteriaceae* in Saudi Arabia. Continued surveillance remains necessary because resistant lineages carrying mobile antimicrobial resistance determinants may persist and disseminate even when their observed frequency is low. The small number of confirmed ESBL-producing isolates also limited formal statistical comparisons among bacterial species, food sources, resistance phenotypes, and genomic determinants.

Future studies should include a greater number of ESBL-producing *Enterobacteriaceae*, including *E. coli*, *Klebsiella*, and *Proteus* spp., to better understand their genetic diversity, resistance determinants, and epidemiological relevance. Thus, to obtain a more comprehensive understanding of ESBL-producing *Enterobacteriaceae* in the food chain, broader surveillance should include a wider range of geographical regions in Saudi Arabia, production systems, and food groups.

Moreover, functional studies are essential to evaluate the expression and phenotypic effects of virulence genes, as genomic identification alone may not accurately represent their biological activity. The genomic analysis did not include contig-level classification of antimicrobial resistance and virulence-associated genes as chromosomal or plasmid-associated. Consequently, the physical location of these determinants and their linkage to the detected plasmid replicons could not be established. Plasmid-replicon detection indicates the presence of particular plasmid families but does not confirm that specific resistance or virulence-associated genes are carried by those plasmids. In addition, the short-read assemblies did not permit complete plasmid reconstruction or confirmation of the physical co-localization and transferability of the detected determinants. Conjugation experiments and other functional transfer assays were not performed; therefore, horizontal gene transfer and conjugative transferability were not demonstrated. Future studies using dedicated genomic-context analysis, long-read or hybrid sequencing, and functional transfer experiments are required to determine the precise location, genetic organization, and potential mobility of the detected genes.

In addition, the absence of contemporaneous isolates from humans, animals, and production environments prevented direct assessment of transmission across One Health sectors. Future surveillance should integrate isolates and metadata from multiple sectors to better investigate possible transmission pathways.

Despite these limitations, the study provides baseline evidence for integrating phenotypic antimicrobial susceptibility testing and whole-genome sequencing into food-chain antimicrobial resistance surveillance in Saudi Arabia. The combined approach enabled the identification of poultry-associated ESBL-producing *Salmonella* Infantis ST32 isolates carrying *blaCTX-M-65* together with multiple resistance determinants, virulence-associated genes, and plasmid replicons. These findings support risk-based monitoring of the poultry-production chain and the prioritization of resistant lineages for further genomic surveillance. They also provide information relevant to food-industry contamination control, future source-attribution investigations, antimicrobial stewardship, and One Health risk assessment.

## 5. Conclusions

This study combined phenotypic screening of 160 foodborne *Enterobacteriaceae* isolates with genomic characterization of confirmed ESBL-producing isolates from Saudi Arabia. ESBL production was uncommon within the examined collection and was concentrated in two poultry-associated *Salmonella* Infantis isolates belonging to ST32 and carrying *blaCTX-M-65* together with multiple additional antimicrobial resistance determinants, virulence-associated genes, and plasmid replicons.

Although the detected genotype and resistance profile have previously been reported, including in Saudi Arabia, the present study provides additional surveillance evidence that clinically important resistance may be concentrated within particular poultry-associated lineages rather than broadly distributed among the examined food isolates. The findings support risk-based monitoring of *Salmonella* Infantis in the poultry-production chain and demonstrate the value of integrating phenotypic antimicrobial susceptibility testing with whole-genome sequencing.

Because the study was based on a limited, archived, and non-random isolate collection, the findings should not be interpreted as nationally representative prevalence estimates or as evidence of a novel transmission pathway. Larger prospective studies incorporating geographically representative sampling, higher-resolution genomic analysis, and human, animal, food, and environmental isolates are required to investigate transmission and the broader epidemiological significance of ESBL-producing *Salmonella* Infantis in Saudi Arabia.

## Figures and Tables

**Table 1:** Distribution of *Enterobacteriaceae* isolates recovered from different food sources included in this study (*n* = 160).

Bacterial Species	No. of Isolates (*n*) (%)	Food Sources
*Escherichia coli O157*	68 (39.5)	Chicken and Beef Meat
*Escherichia coli O145*	5 (2.9)	Chicken and Beef Meat
*Proteus mirabilis*	13 (7.6)	Chicken meat
*Salmonella* spp.	56 (32.6)	Chicken meat
*Klebsiella pneumoniae*	15 (8.7)	Leafy vegetables
*Cronobacter sakazakii*	3 (1.7)	Powdered milk

**Table 2:** Initial phenotypic screening for potential extended-spectrum β-lactamase (ESBL) production among *Enterobacteriaceae* isolates.

Bacterial Species	Total Isolates (*n*)	Screening-Positive Isolates *n* (%)	Screening-Negative Isolates *n* (%)
*Escherichia coli O157*	68	0 (0)	68 (100)
*Escherichia coli O145*	5	0 (0)	5 (100)
*Proteus mirabilis*	13	13 (100)	0 (0)
*Salmonella* spp.	56	53 (94.6)	3 (5.4)
*Klebsiella pneumoniae*	15	0 (0)	15 (100)
*Cronobacter sakazakii*	3	0 (0)	3 (100)

## Data Availability

All sequence data were deposited in NCBI GenBank under BioProject accession numbers PRJNA1432109. The raw reads have been deposited at the Sequence Read Archive (SRA) under accession numbers SRP681057. The complete TORMES bioinformatics analysis report, including assembly statistics, quality metrics, multilocus sequence typing, serotype prediction, antimicrobial resistance genes, virulence-associated genes, plasmid replicons, and other analytical outputs for all sequenced isolates, is provided as Supplementary Report. The raw sequencing reads generated in this study have been deposited in the NCBI Sequence Read Archive (SRA) under BioProject accession PRJNA1432109 (SRA accession SRP681057). The correspondence between laboratory isolate identifiers, sequencing identifiers, BioSample accessions, and SRA accession numbers is provided in Supplementary Table S2.

## Supplementary Materials

The following supporting information can be downloaded at: https://www.mdpi.com/article/10.3390/foods15152585/s1, Table S1: Phenotypic confirmation of ESBL production among Salmonella isolates by clavulanic acid synergy testing; Table S2: Correspondence between laboratory isolate identifiers, sequencing identifiers, BioSample accession numbers, and SRA accession numbers; Figure S1: Comprehensive resistance-associated gene profile of Salmonella Infantis isolates.
