# Supplementary material for: Prevalence and Genomic Insight of Extended-Spectrum β-Lactamase-Producing Enterobacteriaceae (ESBL-E) Isolated from Food Sources in Saudi Arabia
Source: Foods. 2026 Jul 23;15(15):2585. doi: 10.3390/foods15152585 (PMC13464317; doi:10.3390/foods15152585)
Supplement: Supplementary file 1 [file foods-15-02585-s001.zip › foods-4386658-supplementary.pdf]

Supplementary Table S1. *Phenotypic confirmation of ESBL production among* Salmonella *isolates by clavulanic acid synergy testing. Minimum inhibitory concentrations (MICs) of cefotaxime (CTX) and ceftazidime (CAZ) were determined in the presence and absence of clavulanic acid (CLA) according to CLSI guidelines. Fold reduction values were calculated as the MIC of the antibiotic alone divided by the MIC in the presence of clavulanic acid. ESBL production was confirmed when a ≥3 two-fold reduction in MIC was observed with clavulanic acid.*

Isolate	CTX MIC (µg/mL)	CTX + CLA MIC (µg/mL)	CTX fold reduction	CAZ MIC (µg/mL)	CAZ + CLA MIC (µg/mL)	CAZ fold reduction
S53530	32	0.25	128	4	0.5	8
S63589	32	0.25	128	2	0.5	4
S63705	32	0.25	128	2	0.5	4
S64889	32	0.25	128	2	0.5	4
S64911	32	0.25	128	2	0.5	4
S49386	64	0.25	256	4	1	4
S49333	32	0.5	64	4	0.5	8
S49351	64	0.25	256	4	0.5	8
S49350	32	0.25	128	4	0.5	8
S43968	64	0.5	128	8	0.5	16
S41053	64	0.25	256	4	0.5	8
S63596	64	0.5	128	4	0.5	8
S63582	64	0.25	256	4	0.5	8
S433597	64	0.5	128	4	1	4
S193967	32	0.25	128	4	0.5	8

Restricted - مقيد

Supplementary Table S2. *Correspondence between laboratory isolate identifiers, sequencing identifiers, BioSample acces-sion numbers, and SRA accession numbers.*

Laboratory isolate ID	Sequence identifier	Serovar	BioSample	SRA ID	BioProject
**53530**	ESBL_WGS1	*Salmonella Infantis*	SAMN56338991	SRR37469505	PRJNA1432109
**63589**	ESBL_WGS2	*Salmonella Infantis*	SAMN56338992	SRR37469504	PRJNA1432109
**63705**	ESBL_WGS3	*Salmonella Infantis*	SAMN56338993	SRR37469500	PRJNA1432109
**64889**	ESBL_WGS4	*Salmonella Infantis*	SAMN56338994	SRR37469499	PRJNA1432109
**64911**	ESBL_WGS5	*Salmonella Infantis*	SAMN56338995	SRR37469498	PRJNA1432109
**49386**	ESBL_WGS6	*Salmonella Infantis*	SAMN56338996	SRR37469497	PRJNA1432109
**49333**	ESBL_WGS7	*Salmonella Infantis*	SAMN56338997	SRR37469496	PRJNA1432109
**49351**	ESBL_WGS8	*Salmonella Infantis*	SAMN56338998	SRR37469495	PRJNA1432109
**49350**	ESBL_WGS9	*Salmonella Infantis*	SAMN56338999	SRR37469494	PRJNA1432109
**43968**	ESBL_WGS10	*Salmonella Infantis*	SAMN56339000	SRR37469493	PRJNA1432109
**63596**	ESBL_WGS12	*Salmonella Infantis*	SAMN56339001	SRR37469503	PRJNA1432109
**63582**	ESBL_WGS13	*Salmonella Infantis*	SAMN56339002	SRR37469502	PRJNA1432109
**193967**	ESBL_WGS14	*Salmonella Infantis*	SAMN56339003	SRR37469501	PRJNA1432109

Restricted - مقيد

Figure S1. Comprehensive resistance-associated gene profile of *Salmonella* Infantis isolates. The heatmap showing resistance-associated genes identified from whole genome sequencing using the Comprehensive Antibiotic Resistance Database (CARD). Genes include acquired antimicrobial resistance genes as well as other resistance-related determinants, such as multidrug efflux pumps and regulatory genes. Blue cells indicate gene presence, while white cells indicate absence.

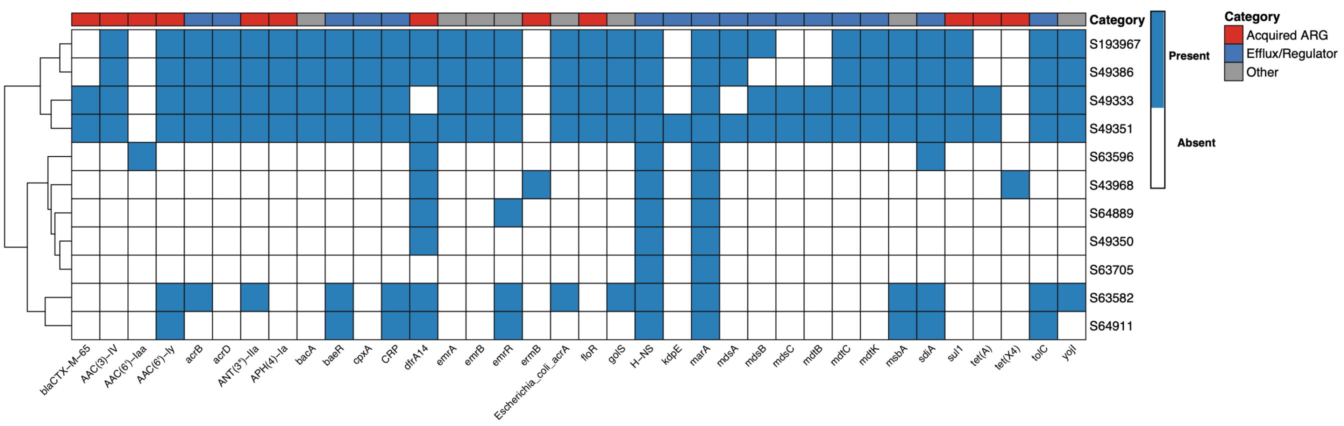

Restricted - مقيد
